# A Novel Ensemble Framework for Multi-Classification of Brain Tumors Using Magnetic Resonance Imaging

**DOI:** 10.3390/diagnostics14040383

**Published:** 2024-02-09

**Authors:** Yasemin Çetin-Kaya, Mahir Kaya

**Affiliations:** Department of Computer Engineering, Faculty of Engineering and Architecture, Tokat Gaziosmanpaşa University, Tokat 60250, Turkey; mahir.kaya@gop.edu.tr

**Keywords:** brain tumor classification, convolutional neural network, deep learning, particle swarm optimization, computer-aided diagnosis

## Abstract

Brain tumors can have fatal consequences, affecting many body functions. For this reason, it is essential to detect brain tumor types accurately and at an early stage to start the appropriate treatment process. Although convolutional neural networks (CNNs) are widely used in disease detection from medical images, they face the problem of overfitting in the training phase on limited labeled and insufficiently diverse datasets. The existing studies use transfer learning and ensemble models to overcome these problems. When the existing studies are examined, it is evident that there is a lack of models and weight ratios that will be used with the ensemble technique. With the framework proposed in this study, several CNN models with different architectures are trained with transfer learning and fine-tuning on three brain tumor datasets. A particle swarm optimization-based algorithm determined the optimum weights for combining the five most successful CNN models with the ensemble technique. The results across three datasets are as follows: Dataset 1, 99.35% accuracy and 99.20 F1-score; Dataset 2, 98.77% accuracy and 98.92 F1-score; and Dataset 3, 99.92% accuracy and 99.92 F1-score. We achieved successful performances on three brain tumor datasets, showing that the proposed framework is reliable in classification. As a result, the proposed framework outperforms existing studies, offering clinicians enhanced decision-making support through its high-accuracy classification performance.

## 1. Introduction

The brain comprises interconnected neurons and is the central nervous system’s paramount component. It oversees and regulates the body’s functions [1]. Brain tumors are masses created by irregular clusters of cells within the brain, and these cells proliferate rapidly and without restraint [2]. Meningioma, glioma, and pituitary tumors are among the most prevalent varieties of brain tumors. Brain tumors can potentially be life-threatening, with World Health Organization reports indicating that 120,000 individuals have succumbed to this condition in recent years. Magnetic resonance imaging (MRI) assists in identifying various brain tumor types [3]. In brain tumor treatment, essential factors include its type, location, and size [1]. The intricate variations within brain tumor cells can complicate determining the tumor type and the suitable treatment strategy, potentially resulting in varying clinician assessments [4]. Therefore, in this study, a computer-aided diagnosis system was developed to classify brain tumor types accurately and quickly from MR images.

Artificial intelligence applications are used in many areas, from cloud computing [5] to disease diagnosis [6,7,8] with medical images. Different methods in the literature exist to analyze images, such as CNN, vision transformers, and capsule networks. While vision transformers require large data sets for training, capsule networks focus more on the part-to-whole relationship. CNN, on the other hand, has many successful applications in the field of medical imaging. When used in concert with pre-trained models, it can effectively capture common features learned across a large data set. Transfer learning is advantageous in terms of overall performance in limited labeled data situations. This study preferred CNN due to its task suitability, practical applicability, and reliability.

Convolutional neural networks (CNNs), a sub-branch of deep learning, have an architecture that can perform end-to-end learning. As we increase the depth and width dimensions in CNN architectures, we encounter overfitting and gradient vanishing problems in datasets with limited labeled data [9]. The gradient vanishing problem is usually solved with residual connections [10]. Complex models with more learning parameters than the amount of data show high performance in the training phase due to overfitting; however, they perform poorly on test data they have not seen before [11]. To avoid overfitting in these models, we usually use regularization techniques such as L2 regularization, dropout, batch normalization, and data augmentation [12,13,14]. In cases where these techniques are insufficient, ensemble learning techniques can be used, combining different features from the dataset using multiple models [15]. The ensemble learning technique can solve the overfitting problem by combining features of different models with different properties from the available dataset. However, the issue of which models to combine and what weight to use still needs to be solved.

This work aims to combine several CNN models with optimal weights using ensemble learning to classify brain tumor types accurately. To overcome the problem of overfitting on limited labeled datasets, we trained and analyzed several models with different architectures on three brain tumor datasets. All layers of these models with different architectures were retrained on the brain datasets and fine-tuned according to the validation datasets. The success order was determined according to their performance on the test datasets. The optimal weights of the five highest-performing models were obtained by the particle swarm optimization (PSO) algorithm [16]. These models are combined with the optimal weights to avoid overfitting and high inter-class similarity. With our proposed method, we achieved successful performances on three brain tumor datasets. Thus, this framework will support clinicians’ decision-making and expedite the diagnostic process.

### 1.1. Motivation

Early identification of the brain tumor type and prompt initiation of treatment are crucial for effective intervention. Automating the classification process with computer-aided systems will reduce the workload of expert clinicians and speed up decision-making processes. Many studies classify brain tumors using CNN architectures. Although existing studies use scratch models, transfer learning, and ensemble techniques, there are some shortcomings in the classification of brain tumors from MR images. These can be listed as follows:

- Existing studies have generally applied the ensemble technique by majority voting on a few predetermined CNN models. To the best of our knowledge, there are no studies in the literature on determining the base models and the weights to which they will contribute.
- Even if the CNN models proposed in existing studies are optimized, they perform limited feature extraction from the dataset. For example, features extracted from a scratch CNN model or a few predetermined CNN models fall into this group. Feature extraction should be diversified with CNN models with different architectures.

The primary motivation of this paper is to attain optimal ensemble performance by utilizing the best base models and introducing a novel weighted method specifically designed for the brain tumor MRI dataset. In this study, an analysis of the accuracy/loss graphs throughout the training and validation phases indicates that the models achieved high accuracy during training. However, advanced approaches are needed for a reliable and better-performing model.

### 1.2. Contributions

In this study, a new weighted ensemble method is proposed for the classification of brain tumors from MR images. The most successful ensemble model is obtained with different models and weights on three publicly available brain tumor datasets. The study offers the following list of contributions:

- We introduce a new ensemble strategy for gathering the best performance. The most appropriate CNN models were iteratively identified and combined with ensemble learning at optimum weights to classify three brain tumor types accurately.
- Utilized a PSO-based algorithm to find the optimum weights that enhance the performance of ensemble CNN models.
- The proposed PSO-Ensemble framework utilizes three different datasets and demonstrates outstanding performance, as supported by extensive experimental results.
- Existing studies have generally not presented the use of their models. The framework proposed in this study is integrated into the online system and available for use (https://ai.gop.edu.tr/bt, accessed on 8 February 2024).

The remaining sections of this study are organized as follows. Section 2 categorizes existing studies that use medical images for disease detection. Section 3 describes the datasets used in the study. Details of the proposed method are given. Section 4 presents extensive experimental studies and results. Section 5 discusses the results compared with the existing work. Finally, Section 6 summarizes the conclusions and future work.

## 2. Related Works

CNNs are widely used for analyzing medical data, such as MRI and X-ray data [17,18]. In medical image classification using CNNs, three primary strategies can be found in the literature. The initial approach involves creating custom CNN models and enhancing their performance through diverse optimization techniques. The second strategy employs transfer learning in conjunction with state-of-the-art CNN models. At the same time, the third approach applies classical machine learning techniques, utilizing CNN models solely for feature extractors.

In the first approach, custom model building, researchers create CNN models from scratch and train the model from start to finish. Custom CNN models require a large amount of labeled data to be trained and are also expected to have a large variety of data. Limited access to labeled data in the medical field is an important limitation to the success of custom models. This may cause overfitting or underfitting problems in the custom models. In addition, determining the optimal depth and width parameters for the CNN architecture is time-consuming.

Numerous studies [19,20,21,22,23,24,25] have explored the creation of custom CNN architectures and enhanced these models through various methodologies for detecting brain tumor types. Ayadi et al. [19] proposed a CNN architecture comprised of ten convolutional layers to classify brain tumors. Raza et al. [20] created an advanced GoogleNet model in their study. The proposed model achieved 99.67% accuracy on a three-class dataset. Khan et al. [21] proposed two models in their study. Model 1 was tested on the Figshare dataset. In Model 2, Model 1 is added to the VGG16 model. Rahman and Islam [22] developed a novel CNN structure in their research. Asif et al. [23] used DenseNet201, DenseNet121, Xception, ResNet152V2, and InceptionResNetV2 architectures by modifying their last layer. The Xception architecture achieved a high accuracy rate of 99.67% on the 3-class dataset. A CNN model created by Saurav et al. [24] uses channel-attention blocks to concentrate on pertinent areas of the image for tumor classification. The selection of the pertinent feature maps is carried out via channel-attention blocks. Akter et al. [25] performed binary classification with a 39-layer model.

To overcome the challenges of developing a custom CNN model, the researchers employed a transfer learning approach. This second approach takes state-of-the-art models trained on large datasets and modifies and adapts their classification layers to the problem at hand. Instead of training the entire model from beginning to end, some layers are frozen. The disadvantages of using transfer learning include the possibility of noises being transferred as features due to limited data and the fact that only one model is used, which limits feature extraction diversity.

Transfer learning and feature extraction methods are also widely used to detect brain tumor types. Deepak and Ameer [26] combined the GoogleNet architecture with a transfer learning approach to extract features from brain MRI images. Alongside the Softmax classifier, the study explored the use of SVM and KNN algorithms. Notably, the KNN algorithm achieved the highest accuracy rate, reaching 98%, with 80% of the dataset allocated for training. Swati et al. [27] tried to achieve high accuracy using AlexNet, VGG16, and VGG19 models with transfer learning. The VGG19 model performed the best, with 94.82% accuracy. Abdelaziz et al. [28] used the ResNet50 model in their study. Mehrotra et al. [29] used various transfer learning architectures. They also utilized various optimizers, including SGDM, Adam, and RMSProp, to improve the models’ success rates. As a result, the AlexNet model achieved a high accuracy of 99.04%. In [30], Rasool et al. used the GoogleNet model for feature extraction and SVM for classification. Badije and Deniz Ülker [31] used the AlexNet model in their study. Alnowami et al. [32] used the DenseNet architecture in their work. Talukder et al. [33] used various transfer learning architectures (DenseNet201, InceptionResNetV2, ResNet50V2, and Xception) in their study. The highest accuracy of 99.68% was achieved with ResNet50V2. Zulfiqar et al. [34] applied a transfer learning-based fine-tuning approach to classify brain tumors into three categories using EfficientNet architectures. Alanazi et al. [35] first developed CNN models consisting of 19, 22, and 25 layers to detect the presence of brain tumors. They performed brain tumor classification using the transfer learning method with the best-performing 22-layer model. Gomez et al. [36] performed a four-class brain tumor type identification study with a 17-layer custom CNN and six pre-trained models, namely EfficientNetB0, InceptionV3, InceptionResNetV2, MobileNetV2, ResNet50, and Xception.

In the third approach, known as ensemble modeling, several architectures are trained concurrently, and the output is combined using various methods (such as feature concatenation and majority voting). Consequently, feature extraction diversity is achieved, in contrast to the transfer learning approach, since features are extracted using multiple architectures. The overfitting issue can be resolved by integrating the features of many models with various attributes from the available dataset. However, there is still a problem with deciding which models to combine and how much weight to use.

Ensemble models have been proposed by some researchers to detect brain tumor types [1,37,38,39,40,41,42]. Aurna et al. [1] proposed a two-stage method for brain tumor classification. They determined the best feature extractors from five pre-trained models, and a new one called Scratched CNN. The top-performing model pairs (EfficientNet-B0, ResNet-50, and scratched CNN) were initially selected and used in the feature extraction stage. The classification was conducted using five algorithms (Softmax, SVM, RF, KNN, and AdaBoost), with Softmax achieving the highest performance. Rezaei et al. [37] combined KNN, weighted kernel width SVM (WSVM), and histogram intersection kernel SVM (HIK-SVM) algorithms with the MODE-based ensemble technique in the classification phase of their study. Noreen et al. [38] proposed two models in their study. In Model-1, the Inception-v3 model was utilized to extract features, while Model-2 employed the Xception model. Then, in both models, Random Forest, Support Vector Machine, and K-Nearest Neighbors algorithms were used for classification using the ensemble technique. In their study, Patil and Kirange [39] combined SCNN and VGG16 models in the feature extraction phase using ensemble learning. Extreme Gradient Boosting, Ada-Boost, and Random Forest (XG-Ada RF) are three high-performance individual machine learning models that Khan et al. [40] suggested as an ensemble for binary classification. Tantel et al. [41] combined five CNN (AlexNet, VGG16, ResNet18, GoogleNet, and ResNet50) architectures with ensemble techniques for binary tumor classification. Features were retrieved for brain tumor classification using several deep learning architectures in the study by Kang et al. [42]. Then, the best three features are combined, and classification is performed with nine different machine learning algorithms. 

Grid search, statistical-based optimization algorithms, and other heuristics were also used to detect brain tumor types. The following are used in brain tumor classification studies: Bayesian optimization algorithm [43], grid search [44], Nonlinear Lévy Chaotic Moth Flame Optimizer (NLCMFO) [45], Combined Political Optimizer [46], Improved Political Optimizer [47], Genetic Algorithm (GA) [48]. 

Evolutionary algorithms were widely used in the optimization of CNN models. The Firefly Optimization Algorithm (FA) [49], Elephant Hearding Optimization Algorithm (EHO), and Hybrid Elephant Hearding Optimization Algorithm (HEHO) [50] were used to optimize the hyperparameters of the CNN. A CNN model based on binary swallow swarm optimization (BSSO) was developed by Kothandaraman [51]. Rammurthy and Mahesh [52] used WHHO, which is an integration of the Whale optimization algorithm (WOA) and the Harris Hawks optimization (HHO) algorithm. Chawla et al. proposed a bat-CNN model in [53]. Sharif et al. [54] used differential evolution and mouth flame optimization algorithms for feature extraction in their study. Xu and Mohammadi [55] used the Mobilenetv2 deep learning model optimized with the innovative meta-heuristic Fox Optimization Algorithm (CFO). 

When the existing studies are examined, there are many studies using scratch models [19,20,21,22,23,24,25], transfer learning [26,27,28,29,30,31,32,33,34,35,36], ensemble learning [1,37,38,39,40,41,42], and different optimization algorithms [4,43,44,45,46,47,48,49,50,51,52,53,54]. Table 1 summarizes related studies on brain tumor classification in terms of method, dataset, classification type, and results. The proposed study optimizes CNN models with different architectures and determines the most successful models. These best CNN models were combined with optimum weights, the ensemble technique was applied, and successful classification performance was obtained.

## 3. Materials and Methods

### 3.1. Dataset

**Figure 1:**
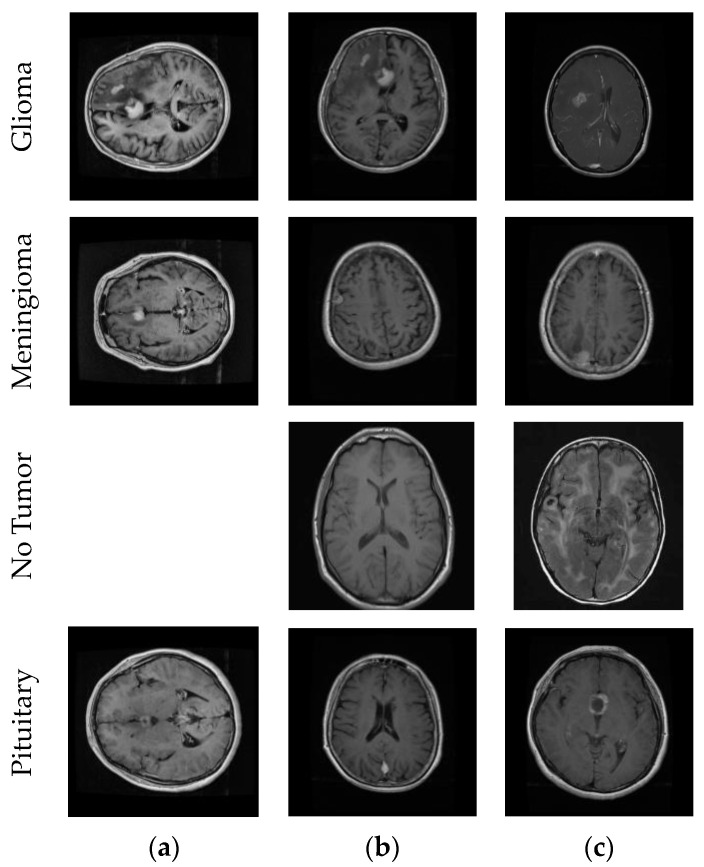
Sample MR images from the datasets. (**a**) Dataset 1; (**b**) Dataset 2; (**c**) Dataset 3.

This study leveraged three datasets for its research purposes. To begin with, dataset 1 [56] is a publicly accessible Figshare brain tumor dataset containing a total of 3064 brain MRIs. This dataset has three distinct classes: glioma, meningioma, and pituitary tumors. Specifically, this dataset comprises 1426 glioma images, 708 meningioma images, and 930 pituitary tumor images. Moving to dataset 2 [57], it is composed of four classes: glioma (926 images), meningioma (937 images), pituitary tumors (901 images), and a category denoting the absence of tumors (500 images). Finally, dataset 3 [58] is also an open-source brain tumor dataset that merges data from three sources: Figshare [56], SARTAJ [57], and Br35H [59], resulting in a total of 7023 brain MRIs. This dataset represents four categories: healthy brain images, meningioma, pituitary, and glioma tumors. Concretely, there are 2000 images of healthy individuals, 1621 glioma images, 1645 meningioma images, and 1757 of pituitary tumors. Figure 1 shows example MR images from the datasets.

**Figure 2:**
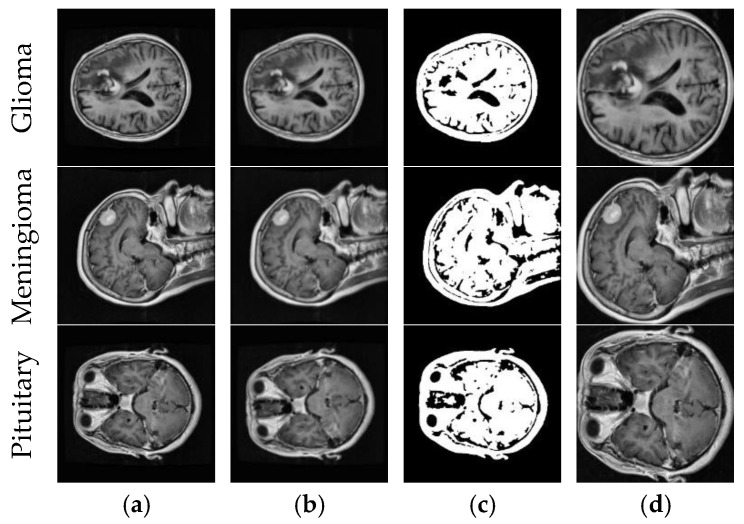
Brain tumor types and the process of the image segmentation algorithm. (**a**) Original image; (**b**) Gaussian blur with a 9 × 9 kernel; (**c**) binary image-otsu thresholding; (**d**) final image.

We divided the datasets into train, validation, and test. First, we split the datasets into 80% train and 20% test. Then, we split 10% of the training datasets into validation. Figure 2 shows example MR images of brain tumor types and the process of the image segmentation algorithm.

The MRI images were first preprocessed. In Figure 2, a noise outside the brain region was removed. For CNN architectures to focus only on the brain region, we first applied Gaussian blur with the 9 × 9 kernel and then applied Otsu thresholding to extract the binary image. The brain region’s contours were detected in the binary image, and brain region segmentation was performed based on the extreme points of the largest contour in all directions. Thus, CNN architectures will only operate within the brain region in real-time applications.

### 3.2. Transfer Learning

CNN architectures are usually built sequentially, combining convolution, pooling, and fully connected layers. With CNNs, feature vectors are automatically obtained from the input images during the training phase, and classification is performed. In the training phase, learning is achieved by updating the filter weights in the convolution layer and the weights of the fully connected layer according to the training error. The back-propagation algorithm is generally implemented to update weights [8,60].

With transfer learning, CNN models trained on large datasets, such as ImageNet, are retrained on new datasets by preserving the weights of the parameters in the filters and fully connected layers [61]. In CNN models, the first layers usually learn basic features such as lines, edges, and color blobs, while the last layers learn more detailed forms relevant to the problem at hand [62]. Therefore, in classical image classification problems, convolution layers are usually frozen in the training phase and are not trained on the new dataset, and successful results are obtained by updating the parameters in the fully connected layers. However, the training should also include convolution layers in medical images. Since the process of labeling medical images by an expert is costly and, in some cases, there needs to be more diseased images, disease detection from medical images is usually faced with the problem of limited labeled data. Although the transfer learning method gives successful results in these cases, there is usually an overfitting problem in the training phase. In this study, CNN models are constructed by preserving the previous parameter values until the last convolution layer. After the final convolution layer, global average pooling and flattening layers are analyzed separately. The number of fully connected layers, neurons in each layer, and dropout rates were optimized. In the last layer, a layer with four neurons was added, along with the Softmax activation function. The first layers were frozen in the training phase, and training was performed. Finally, in all datasets, the parameters in all layers of the CNN models were updated, and training was completed.

### 3.3. Proposed Framework

**Figure 3:**
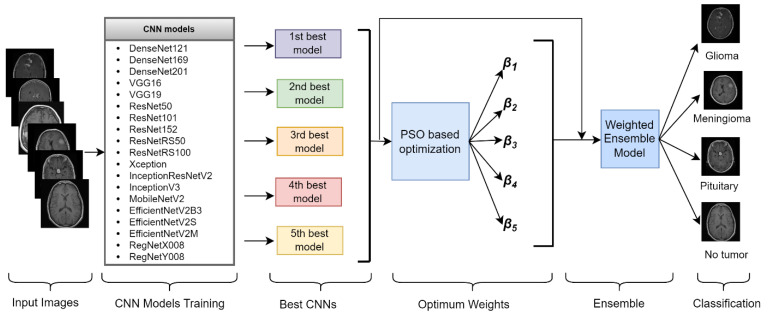
General structure of the proposed framework.

Several CNN models with different architectures were retrained on the brain tumor dataset with transfer learning and fine-tuning (see Figure 3). In these models, various hyperparameters were optimized with grid search to determine the most successful models. Table 2 summarizes the optimized hyperparameters and their values. CNN models are constructed by preserving the previous parameter values until the last convolution layer. After the final convolution layer, global average pooling and flattening layers were analyzed separately. The last layer was added with three or four neurons using the Softmax activation function. CNN models were retrained for 50 epochs. The study employed a batch size of 16. During the training phase, all layers of the models in the study were retrained. Three datasets were used in this study. The five best-performing models on each dataset were identified, and their performance on the test dataset was found using ensemble learning. The PSO-based algorithm determined the weights of the five models for ensemble learning.

The PSO algorithm, one of the algorithms based on swarm intelligence, was proposed by Kennedy and Eberhart [16]. The algorithm consists of a swarm and individuals (solutions) called particles within the swarm. The algorithm starts with a set of randomly generated particles, and the particles are updated at each iteration to determine the optimal value. In each iteration, each particle is updated according to two values. The first one is *X_i_*_,*pbest*_, which is the best fitness value that a particle has found so far. The second value is the best fitness value obtained so far by any particle in the swarm, called *X_gbest_*. These values are also stored in memory for later use. After finding the best cases of both values, the velocities and positions of the particles are updated according to the formulas shown in Equations (1) and (2).

(1) $$V_{i,new}=\omega \times V_{i,j}+c_{1}\times r_{1}\times \left(X_{i,p_{best}}-X_{i,j}\right)+c_{2}\times r_{2}\times \left(X_{g_{best}}-X_{i,j}\right)$$

(2) $$X_{i,new}=X_{i,j}+V_{i,new}$$

In Equation (1), *c*_1_ and *c*_2_ are the acceleration factors and provide the correct orientation of *X_i_*_,*pbest*_, and *X_g_*__*best*_. *C*_1_ is guided by the particle’s own experience, and *c*_2_ is guided by the experience of other particles in the swarm. Random numbers are assigned to *r*_1_ and *r*_2_ as coefficients, and these values are updated in every iteration. Both *r*_1_ and *r*_2_ coefficients are confined to the range of 0 to 1. The inertia weight *ω* is typically chosen to vary between 0.1 and 1. In the PSO algorithm, cognitive weight (*c*_1_) and social weight (*c*_2_) were selected as 1.5. The inertia weight was chosen as 0.7.

A weight (*β_i_*) was assigned to each model, and this weight was estimated using the PSO-based algorithm as detailed in Algorithm 1. We calculate prediction probabilities (*P_i_*) for each model and multiply these predictions by their respective weights (*β_i_*) to determine the final probabilities (*y_pred_*) for classification in Equation (3). *Y_i_* is ground truth (correct) labels. The log loss or objective function is presented in Equation (4). The sum of the weights assigned to each model should be 1, as shown in Equation (5).

(3) $$y_{pred}=\sum_{i=1}^{M}P_{i}\beta_{i},\;M\;\mathrm{denotes}\;\mathrm{the}\;\mathrm{number}\;\mathrm{of}\;\mathrm{models}.$$

(4) $$\mathrm{Loss}=-\frac{1}{N}\sum_{i=1}^{N}\left[y_{i}\times \log\left(y_{pred}\right)+\left(1-y_{i}\right)\times \log(1-y_{pred})\right]$$

(5) $$\sum_{i=1}^{M}\beta_{i}=1,$$

**Algorithm 1** PSO-based weighted ensemble learning algorithmObtain prediction probabilities (P_i_) for each model; initial values of β_i_ are determined randomly for each particle, number of particles:= 100, maxIteration: = 1000 **while** i < maxIteration             **for** particle **in** swarm **do**:                    **for** m **in** models **do**:                            #Calculate final probabilities via Equation (3)                             newPredictions += particle[m] × modelPredictions[m]                  #Calculate objective value (loss) via Equation (4)                 loss_score = log_loss(y, newPredictions)                 results.append(loss_score)              **end for**             **for** j **in** swarm **do**                  **if** results[j] < individualBestResult[j] **then**                           individualBestResult[j]: = results[j]                 **end if**             #Find minimum objective value and β_i_ in particles             **if** min(results) < bestGlobalObjectiveValue **then**                            bestGlobalObjectiveValue: = min(results)                            bestβ_i_: = β_i_             **end if**             Update β_i_ in each particle according to Equations (1) and (2)             Adjust β_i_ in each particle to satisfy Equation (5)             i: = i + 1 **end while**

Figure 3 shows the general structure of our proposed framework. After identifying the most successful CNN models on a dataset, the optimum weights for these models are determined iteratively. When the optimum weights of the ensemble model are determined, the classification phase is started.

### 3.4. Performance Metrics

The performance of the proposed framework is assessed using the following metrics: area under the curve (AUC), recall, accuracy, precision, and F1-score. The AUC score assesses the model’s capacity for class discrimination [63]. The formulas for accuracy, F1-score, precision, and recall metrics calculated from the confusion matrix are presented in Equations (6)–(9) [64].

(6) $$Accuracy=\frac{TP+TN}{\left(TP+TN+FP+FN\right)}$$

(7) $$Precision=\frac{TP}{TP+FP}$$

(8) $$Recall=\frac{TP}{TP+FN}$$

(9) $$F1Score=2\times \frac{Recall\times Precision}{Recall+Precision}$$

## 4. Results

In this study, several state-of-the-art CNN models with different architectures were trained with transfer learning on three brain tumor datasets. CNN models with different architectures can extract various features of the dataset. Since ResNet and DenseNet architectures solve the vanishing gradient problem with residual connections, deeper architectures can usually be defined. The general disadvantage of these architectures is the overfitting problem in the case of limited labeled data. Although many versions of EfficientNet and RegNet architectures exist, the selected architectures generally perform better. The calculations and processes were executed on a standard PC configuration comprising 16 GB of RAM, an NVIDIA GeForce GTX 1080 Ti GPU boasting 11 GB of memory, and an Intel i5-8400 processor.

Table 3 shows the accuracy and F1-score, while Table 4 displays the precision, recall, and AUC values of CNN models on three datasets. In this study, the models were trained five times, and the average values of the trained models on the test dataset are given. Since Dataset 1 and Dataset 2 have a limited number of labeled data, they have limited performance compared to Dataset 3. In DenseNet architectures, data from a convolution layer block is combined with feature map values from all subsequent layers, which generally leads to better performance. In general, deep learning models need many labeled images in the training phase to avoid overfitting and extract general statistical patterns. Since there is enough labeled data in Dataset 3, the models performed better. With transfer learning and fine-tuning, many hyperparameters of CNN models were optimized. In the training phase, many hyperparameters with different values (see Table 2) were optimized with GridSearch.

Table 5 shows the weight ratios of the five CNN models that perform best with the PSO algorithm for three datasets in ensemble learning. Using PSO optimization according to Algorithm 1, the best-performing model weight ratios were found iteratively on the test dataset. CNN models with different architectures can often extract different features from the dataset. Combining these models with optimal weights is essential to improving their performance on the test dataset. The accuracy values with weighted ensemble learning on the datasets were 99.35% for Dataset 1, 98.77% for Dataset 2, and 99.92% for Dataset 3. When these results are compared with the accuracy of the individual CNN models, there is a performance improvement. Moreover, the weighted ensemble learning model produced more stable results.

**Figure 4:**
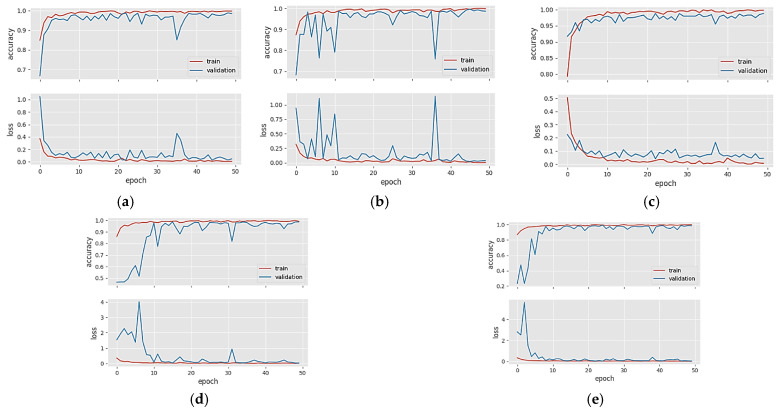
Accuracy/loss plots of five different CNN models in Dataset 1: (**a**) DenseNet121, (**b**) DenseNet201, (**c**) EfficientNetV2S, (**d**) ResNet50, and (**e**) ResNet101.

Figure 4 shows the accuracy/loss graphs of five different CNN models in Dataset 1. The training accuracy line shows an upward trend in the training phase as learning occurs over the epochs. If overfitting or memorization does not occur during the training phase, the validation accuracy line in the validation data will continue to overlap or be parallel with the training accuracy line. When CNN models become overfitted after a certain epoch in the training phase, the validation curve starts to decrease after this epoch. Since we try to optimize the models with the number of neurons in the fully connected layer and the dropout rate during the training phase, the CNN models generally avoid falling into an obvious overfitting state. When the loss graphs are analyzed, the training and validation loss curves decrease throughout the epochs as learning occurs in the training phase. However, in the case of overfitting after a certain epoch in the training phase, the validation loss curve will continue to increase after this epoch. Figure 4a–e shows the accuracy and loss plots of the DenseNet121, DenseNet201, EfficientNetV2S ResNet50, and ResNet101 models in Dataset 1, respectively. Since the training and validation graphs in Figure 4 overlap at several points throughout the epochs, we can say there is no overfitting in Dataset 1.

**Figure 5:**
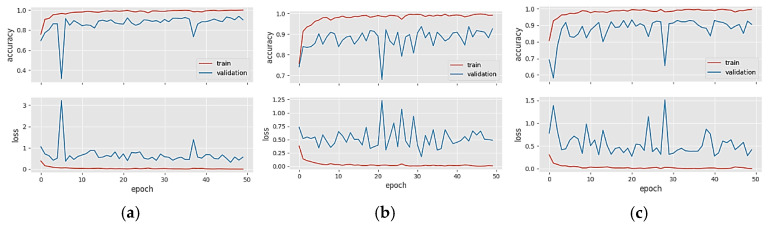

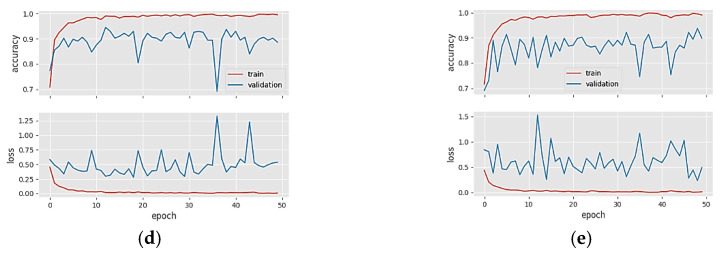
Accuracy/loss plots of five different CNN models in Dataset 2: (**a**) DenseNet121; (**b**) DenseNet169; (**c**) DenseNet201; (**d**) InceptionResNetV2; (**e**) ResNetRS100.

Figure 5a–e shows the accuracy and loss graphs of the DenseNet121, DenseNet169, DenseNet201, InceptionResNetV2, and ResNetRS100 models in Dataset 2, respectively. When the graphs in Figure 5 were examined, we saw that the validation accuracy line followed the train accuracy line from below. Still, the gap between them indicates that the models are in a slightly overfitting situation. For Dataset 2, the data in the training phase needs to be increased.

**Figure 6:**
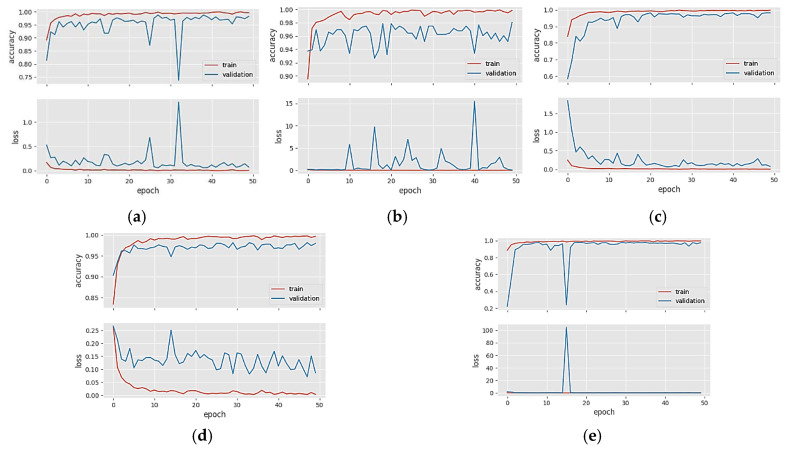
Accuracy/loss plots of five different CNN models in Dataset 3: (**a**) DenseNet201; (**b**) InceptionResNetV2; (**c**) MobileNetV2; (**d**) RegNetX008; (**e**) ResNet101.

Figure 6a–e shows the accuracy and loss graphs of the DenseNet201, InceptionResNetV2, MobileNetV2, RegNetX008, and ResNet101 models in Dataset 3, respectively. When the graphs in Figure 6 were analyzed, we could see that the train and validation accuracy curves overlap at many points and move upwards. CNN models did not fall into overfitting or memorization in Dataset 3.

In this study, when the curves of the accuracy/loss graphs in the training and validation phases were examined, the models exhibited high accuracy in the training phase. Although they partially avoided overfitting with regularization techniques, the models could not reach the desired generalization capacity. In these cases, the ensemble technique should be used, as it will both provide feature diversity and emphasize the strengths of different models.

**Figure 7:**
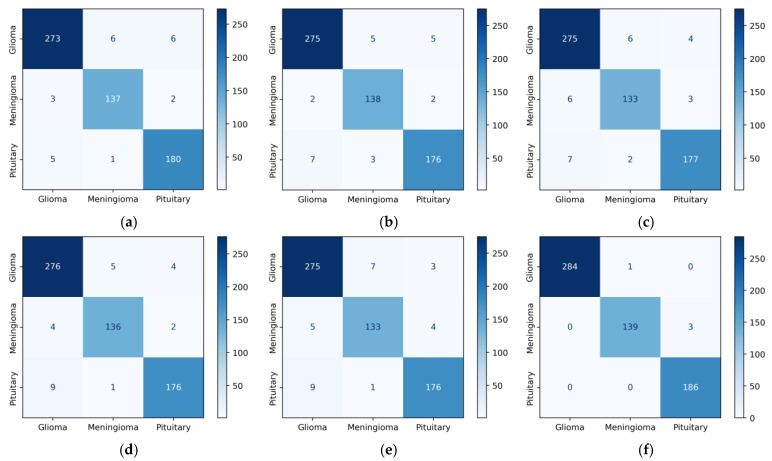
Confusion matrices of CNN models for Dataset 1: (**a**) DenseNet121, (**b**) DenseNet201, (**c**) EfficientNetV2S, (**d**) ResNet50, (**e**) ResNet101, and (**f**) proposed model.

Figure 7a shows the three-class confusion matrix values of the DenseNet121 model in Dataset 1. Horizontal values (rows) represent actual values. Vertical values (columns) show the predicted values of the model. When Figure 7a was examined, the model correctly classified 273 out of 285 glioma images. This value is true positive (TP) for the glioma class. In the first row, six images with a true label of glioma were misclassified as meningioma and the other six as pituitary. The 12 (6 + 6) incorrectly classified images give a false negative (FN) value. When the column values for glioma were analyzed, three images with the correct label, meningioma, and five images with the correct label, pituitary, were incorrectly predicted as glioma. These eight (3 + 5) images represent the false positive (FP) value for the glioma class. In Figure 7, among the base models, the fewest errors were observed in Densenet121, and the most errors were observed in ResNet101. When the proposed model is analyzed in Figure 7f, three images with the real label of meningioma are incorrectly predicted as pituitary. The number of meningioma and pituitary images can be increased in this dataset.

**Figure 8:**
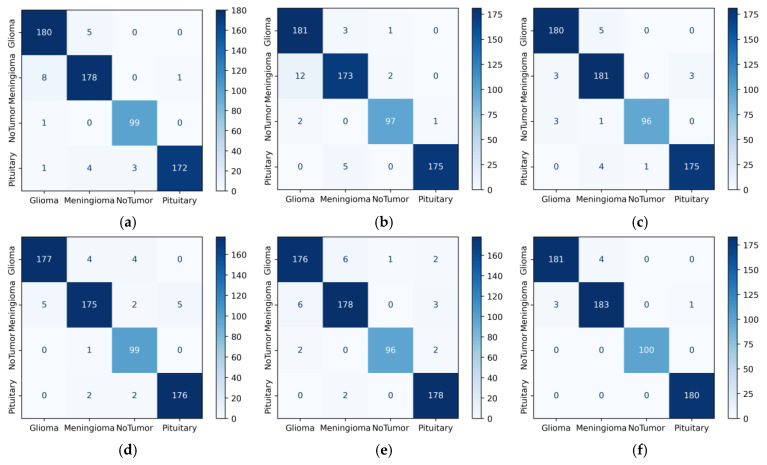
Confusion matrices of CNN models for Dataset 2: (**a**) DenseNet121, (**b**) DenseNet169, (**c**) DenseNet201, (**d**) InceptionResNetV2, (**e**) ResNetRS100, (**f**) proposed model.

Figure 8c shows the confusion matrix values of the DenseNet201 model in Dataset 2. The second row of the confusion matrix in Figure 8c shows that the model correctly predicted 181 of the 187 meningioma tumor images. This value is true positive for the meningioma class. Three images with the true label meningioma were incorrectly predicted as glioma and another three as pituitary. In total, these six (3 + 0 + 3) misclassified images were false negative. When the meningioma column values in Figure 8c are analyzed, five images with the true label glioma, one image with no tumor, and four images with pituitary were incorrectly predicted as meningioma. In total, these ten (5 + 1 + 4) values were false positive. Among the base models, the fewest errors were observed in DenseNet201, and the most errors were observed in DenseNet169. In general, when we look at Figure 8, we can say that the models have difficulties due to the inter-class similarity between Glioma and Meningioma.

**Figure 9:**
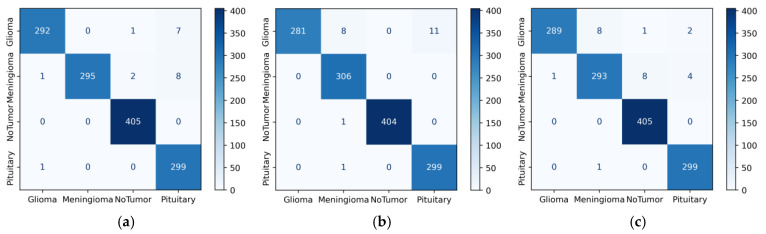

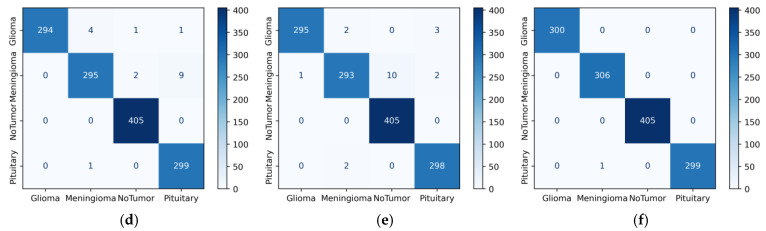
Confusion matrices of CNN models for Dataset 3 (**a**) DenseNet201; (**b**) InceptionResNetV2; (**c**) MobileNetV2; (**d**) RegNetX008; (**e**) ResNet101, (**f**) proposed model.

When we examine the fourth row in Figure 9d (RegNetX008), 299 of the total 300 pituitary images were correctly predicted. In addition, one pituitary was incorrectly predicted as meningioma. This one value gives a false negative value for the pituitary class. When we look at the column values in the pituitary class, one glioma and nine meningioma images were incorrectly predicted as pituitary. These ten values show a false positive value for the pituitary class. Among the base models, the fewest errors were observed in RegNetX008, and the most errors were observed in MobileNetV2. Figure 9f shows the confusion matrix values of the proposed model in Dataset 3. In Figure 9f, we can see that the proposed model performs very well due to the sufficient data in Dataset 3. In addition, when examining the models in all three datasets, DenseNet121, DenseNet201, and InceptionResnetV2 models can be selected as base models.

Table 6 compares our proposed weighted ensemble model with existing studies in the literature regarding accuracy and F1-score measures. Some studies proposed a classification model with only three classes (Glioma, Meningioma, and Pituitary) in the Figshare database instead of the four-class dataset. Our proposed new ensemble model outperforms all existing studies.

CNNs are described as black-box models and do not explain the reason for the classification decision [65]. This prevents interpretation of the results [66]. Since CNN-based state-of-the-art models were used in this study, the interpretability of the results could be improved. To make the decision-making process of CNN models more explicit, the gradient-weighted class activation mapping (GradCAM) technique was utilized [67]. GradCAM is a technique that aids in locating an input image’s crucial regions for predictions, enhancing CNN models’ transparency [68].

**Figure 10:**
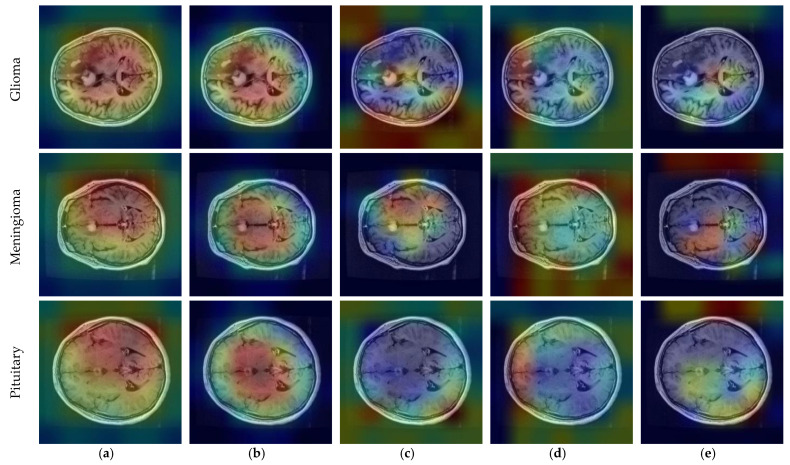
CNN outputs visualized on a heat map with Grad-CAM for Dataset 1: (**a**) DenseNet121, (**b**) DenseNet201, (**c**) EfficientNetV2S, (**d**) ResNet50, and (**e**) ResNet101.

**Figure 11:**
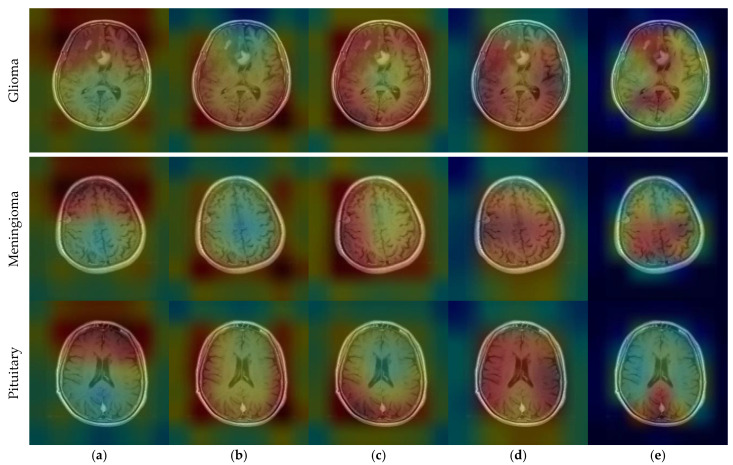
CNN outputs visualized on a heat map with Grad-CAM for Dataset 2: (**a**) DenseNet121, (**b**) DenseNet169, (**c**) DenseNet201, (**d**) InceptionResNetV2, and (**e**) ResNetRS100.

**Figure 12:**
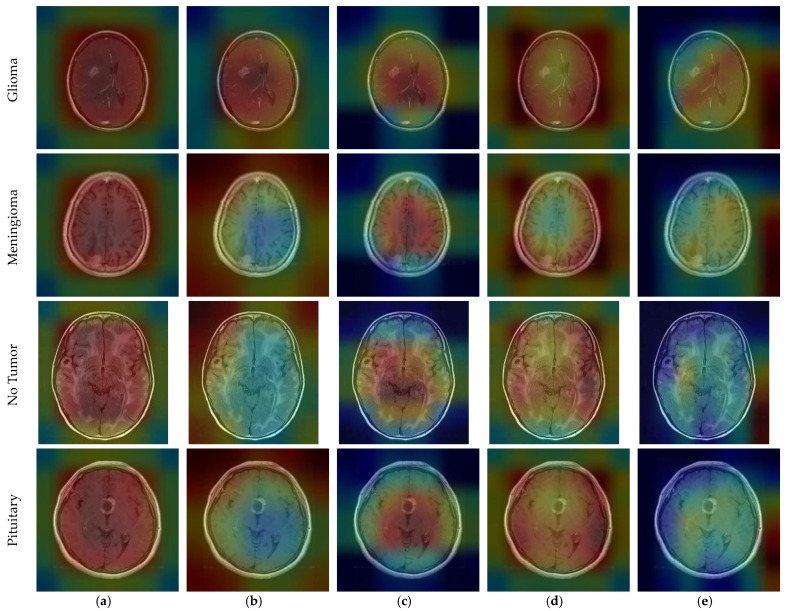
CNN outputs visualized on a heat map with Grad-CAM for Dataset 3: (**a**) DenseNet201; (**b**) InceptionResNetV2; (**c**) MobileNetV2; (**d**) RegNetX008; (**e**) ResNet101.

CNN outputs visualized on a heat map with Grad-CAM for Dataset 1, Dataset 2, and Dataset 3 are represented in Figure 10, Figure 11 and Figure 12, respectively. The original images are overlaid with a color spectrum ranging from blue to red, where the red regions indicate the dominant focus during model predictions. The sample images in Figure 1 were used as the original images in the Grad-CAM application. Grad-CAM analyses of the models in the ensemble framework are presented for three data sets. Figure 10 shows that while all models focus on the brain, different models may focus on different regions of the same MRI image. This trend also helps to increase feature diversity. This can be considered an indicator of better performance with ensemble learning.

**Figure 13:**
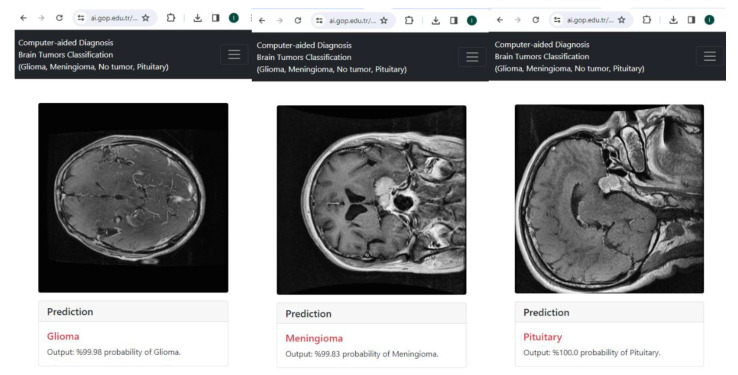
End-to-end real-time web-based system for multi-classification of brain tumors.

Figure 13 shows the real-time implementation of the proposed framework for multi-classification of brain tumors. Furthermore, it is important to highlight that existing research findings have not been completely incorporated into a live system [69]. To fill this gap, the suggested method has been applied to an online system in real-time to showcase its effectiveness and simplicity for physicians to employ.

## 5. Discussion

CNN models, which are used to create the model that will extract features from the raw image in the training phase, make successful classifications with these models after the training is over. CNN models learn the statistical patterns of each class in the data during the training phase. In order for the models to be successful or to generalize the dataset, a large amount of data is required. Databases created with medical images usually have a limited amount of labeled data. When training with limited labeled medical images, it becomes crucial to avoid overfitting. In CNN models, the first layers learn general features such as lines, edges, and color blobs, while later layers learn more complex structures specific to the dataset. With transfer learning, using the filter weights of state-of-the-art CNN models that have been previously trained on large datasets and retraining them on the new dataset at hand can be a solution to the limited labeled dataset problem. These models with different architectures often suffer from overfitting. Successful results can be achieved with ensemble learning, which is based on combining the strengths of different CNN models. When the loss/accuracy graphs of different models in the training and validation phases are examined, it is seen that the validation accuracy graphs follow the training accuracy graphs from below. In this case, since the models cannot generalize fully, they do not reach the desired learning capacity. This study shows that we can overcome this situation with the proposed ensemble technique.

We provide a solution to the problem of which models to combine with ensemble learning and at what weight ratio. With the framework we developed in this study, the most successful CNN models were determined by transfer learning and fine-tuning on a dataset. The optimal ensemble learning weight ratios of the most successful CNN models were found with a PSO-based algorithm. Existing studies usually combine pre-selected CNN models with ensemble learning without finding the optimal weights. With this framework, different CNN models were identified for ensemble learning on three datasets and combined with optimal weights to achieve the highest performance. This framework will contribute to the decision-making process of clinicians and has practical use.

In the diagnosis of brain tumors, studies have been carried out using models from scratch [19,20,21,22,23,24,25], transfer learning [26,27,28,29,30,31,32,33,34,35,36], and ensemble learning [1,37,38,39,40,41,42] techniques. Ayadi et al. [19] performed a brain tumor diagnosis with a scratch model. The model includes 10 consecutive convolutional and batch normalization layers. With the proposed model, an accuracy rate of 94.74% was achieved. Deepak and Ameer [26] used GoogleNet architecture with a transfer learning method. In the study, the best accuracy rate (97.17%) for brain tumor classification was obtained with the KNN algorithm. Aurna et al. [1] investigated the best architectures for ensemble models in brain tumor diagnosis and found that EfficientNet-B0, ResNet-50, and proposed scratch CNN models performed best. They achieved the best accuracy rate (98.96%) by using the two-stage ensemble model and the Softmax classification algorithm. In scratch CNN models, even if the models can be improved by hyperparameter optimization, there is usually an overfitting problem in the training phase due to limited labeled data. The use of pre-trained models on large datasets with transfer learning also provides a partial solution to the problem of limited labeled data; however, deep and complex models also suffer from overfitting, and only one CNN model may be insufficient to learn different features on limited and non-diverse datasets. In Table 6, the best results of existing studies in Datasets 1, 2, and 3 are 98.7%, 95.71%, and 98.96%, respectively. Our proposed method obtained better results than the existing studies in all datasets.

## 6. Conclusions

Detecting brain tumor types in MRI images using computer-aided systems and promptly initiating the appropriate treatment process is paramount. Although CNN models are widely used in disease detection from medical images, they often face the problem of overfitting when training on limited labeled data and data with high inter-class similarity. By employing diverse CNN models with varying architectures and utilizing transfer learning and the ensemble method, we enhance the breadth of feature extraction within the dataset, effectively addressing the overfitting issue. With the framework we developed in this study, we train the CNN models with different architectures on a dataset and determine the best-performing models. Combining these models with a PSO-based algorithm and ensemble method with optimum weights, we detected brain tumor types with high accuracy. We trained the framework on three brain tumor datasets, identified the best CNN models for each dataset, and determined their optimal weights. We obtained 99.92% accuracy and a 99.92% F1-score on the test data of the Dataset 3. The proposed model outperformed the existing studies. We achieved successful performances with our proposed framework on all three brain tumor datasets, which shows that the proposed framework is consistent in brain tumor classification. It contributes to the automatic detection of brain tumor types and doctors’ decision-making processes. Different CNN models will be added to future studies. In addition, this model is planned to be used in other datasets. This research brings numerous advances in the use of deep learning models to classify brain tumors, but it also has some limitations. Data preprocessing was performed prior to training the models with the MRI images in the datasets. One of the study’s shortcomings is the lack of documentation of the model training phase using the original, non-preprocessed images in the datasets. Future research will address this constraint by investigating the role of data preprocessing in the success of brain tumor diagnosis.

## Figures and Tables

**Table 1:** Summary of related studies.

Method	Reference	Year	Dataset	Classification Type	Accuracy (%)
Scratch Model	Ayadi et al. [19]	2021	Figshare MRI	Multi	94.74
			Radiopaedia		93.71
			Rembrandt		95
	Raza et al. [20]	2022	CE-MRI	Multi	99.67
	Khan et al. [21]	2022	Figshare MRI	Multi	97.8
			Harvard Medical	Binary	100
	Rahman and Islam [22]	2023	Figshare MRI	Multi	97.60
			Kaggle-Nickparvar		98.12
	Asif et al. [23]	2023	Figshare MRI	Multi	99.67
			Kaggle -Sartaj		95.87
	Saurav et al. [24]	2023	BT-Small-2C	Binary	96.08
			BT-Large-2C		99.83
			BT-Large-3C	Multi	97.23
			BT-Large-4C		95.71
	Akter et al. [25]	2024	Dataset-a	Binary	96.7
			Dataset-b		89.4
			Dataset-c		97.7
			Dataset-d		95.2
			Merged Dataset-1		98.7
			Merged Dataset-2		97.6
Transfer Learning	Swati et al. [27]	2019	CE-MRI	Multi	94.82
	Deepak and Ameer [26]	2019	Figshare MRI	Multi	97.1
	Abdelaziz et al. [28]	2020	CE-MRI	Multi	99
	Mehrotra et al. [29]	2020	TCIA	Binary	99.04
	Rasool et al. [30]	2022	Kaggle-Sartaj	Multi	98.1
	Badjie and Deniz Ülker [31]	2022	BraTS2020	Binary	99.62
	Alnowami et al. [32]	2022	Dataset-1	Multi	72.10
			Dataset-2		87.02
			Dataset-3		96.52
	Talukder et al. [33]	2023	Figshare MRI	Multi	99.68
	Zulfiqar et al. [34]	2023	Figshare MRI	Multi	98.86
	Alanazi et al. [35]	2022	Br35H	Binary	99.33
			Kaggle-Sartaj	Multi	96.90
			Figshare MRI	Multi	95.75
	Gomez et al. [36]	2023	Kaggle-Nickparvar	Multi	97.12
Ensemble Learning	Rezaei et al. [37]	2020	MRI Dataset	Multi	92.46
	Noreen et al. [38]	2021	MRI dataset	Multi	94.34
	Patil and Kirange [39]	2023	Figshare MRI	Multi	97.77
	Aurna et al. [1]	2022	Figshare MRI	Multi	99.13
			Kaggle-Sartaj	Multi	98.96
	Kang et al. [42]	2021	Kaggle-Sartaj	Multi	93.72
	Khan et al. [40]	2023	Figshare MRI	Binary	95.4
	Tantel et al. [41]	2023	T1W	Binary	94.75
			T2W		97.98
			FLAIR		98.88
With the help of Optimization Algorithms	Ait-Amou et al. [43]	2022	Figshare MRI	Multi	98.70
	Devi [44]	2021	Kaggle-Sartaj	Multi	90.25
	Dehkordi et al. [45]	2022	BRATS 2015	Multi	97.4
	Bashkandi et al. [46]	2023	Br35H	Binary	97.09
	Wu and Sen [47]	2023	Figshare MRI	Multi	95.98
	Anaraki et al. [48]	2019	IXI, REMBRAND, TCGA-LGG	Multi	90.9
			Figshare MRI	Multi	94.2
	Bacanin et al. [49]	2021	IXI, REMBRANDT, TCGA-GBM, TCGA-LGG	Multi	93.3
			Figshare MRI	Multi	96.5
	Bezdan et al. [50]	2021	IXI, REMBRANDT, TCGA-GBM, TCGA-LGG	Multi	94.50
	Kothandaraman [51]	2023	Figshare MRI	Multi	96.125
	Rammurthy and Mahesh [52]	2022	BRATS	Multi	81.6
			SimBRATS		81.6
	Chawla et al. [53]	2022	Figshare MRI	Multi	99.5
	Sharif et al. [54]	2022	BRATS 2013	Multi	99.06
			BRATS 2015		98.76
			BRATS 2017		98.18
			BRATS 2018		94.6
	Xu and Mohammadi [55]	2024	Figshare MRI	Multi	97.32

The numbers and lowercase letters (1, 2, 3 and a–d) here indicate different datasets in the related articles.

**Table 2:** Optimized hyperparameters.

Hyperparameter	Values
Number of fully connected layers	1, 2, 3
Number of neurons in the fully connected layer	64, 128, 256, 512, 1024
Dropout rate	0, 0.1, 0.2, 0.3, 0.4, 0.5, 0.6
Optimizer	Adam, SGD
Learning rate	0.001, 0.0001

**Table 3:** Accuracy and F1-score performance metrics of the state-of-the-art CNN models on three datasets.

CNN Models	Dataset 1 (DS1)		Dataset 2 (DS2)		Dataset 3 (DS3)	
	Accuracy (%)	F1-Score (%)	Accuracy (%)	F1-Score (%)	Accuracy (%)	F1-Score (%)
DenseNet121	96.25	96.18	96.47	96.66	96.65	96.61
DenseNet169	94.13	93.88	96.01	96.15	97.64	97.48
DenseNet201	96.08	95.96	96.93	97.01	98.48	98.38
VGG16	91.52	90.64	82.06	81.88	97.18	96.97
VGG19	94.62	94.11	94.79	94.58	96.91	96.75
ResNet50	95.92	95.86	95.09	95.17	98.14	97.99
ResNet101	95.27	95.07	94.33	94.51	98.47	98.45
ResNet152	93.56	93.46	92.33	92.82	97.66	97.62
ResNetRS50	93.15	92.79	95.55	95.67	97.64	97.44
ResNetRS100	95.19	95.04	96.32	96.46	97.71	97.61
InceptionResNetV2	94.37	94.19	96.17	96.12	98.44	98.28
InceptionV3	94.54	94.41	95.39	95.55	98.09	97.97
Xception	93.47	93.11	95.39	95.55	97.79	97.73
MobileNetV2	90.22	90.59	93.87	94.05	98.09	98.02
EfficientNetV2B3	88.25	87.76	93.40	93.57	97.86	97.74
EfficientNetV2S	95.43	95.22	93.63	93.62	97.56	97.39
EfficientNetV2M	88.01	87.71	95.09	95.09	95.50	95.32
RegNetX008	94.69	94.43	94.94	94.91	98.63	98.54
RegNetY008	95.11	94.98	95.86	95.84	97.18	97.00

**Table 4:** Precision, recall, and AUC performance metrics of the state-of-the-art CNN models on three datasets.

CNN Models	Dataset 1 (DS1)			Dataset 2 (DS2)			Dataset 3 (DS3)		
	Precision (%)	Recall (%)	AUC (%)	Precision (%)	Recall (%)	AUC (%)	Precision (%)	Recall (%)	AUC (%)
DenseNet121	96.01	96.41	97.24	96.60	96.76	97.78	97.21	96.39	97.61
DenseNet169	94.05	93.92	95.48	96.21	96.14	97.39	97.66	97.42	98.32
DenseNet201	95.84	96.10	97.02	97.21	96.83	97.88	98.49	98.35	98.93
VGG16	90.39	90.97	93.35	81.82	81.99	87.96	97.12	96.93	98.0
VGG19	95.20	93.27	95.09	94.60	94.60	96.42	97.0	96.75	97.87
ResNet50	95.99	95.75	96.70	94.77	95.66	97.01	98.14	97.96	98.68
ResNet101	95.22	94.93	96.22	94.5	94.76	96.42	98.56	98.35	98.92
ResNet152	92.91	94.48	95.71	93.88	92.83	95.09	97.85	97.46	98.33
ResNetRS50	92.83	92.85	94.66	95.45	95.98	97.24	97.68	97.42	98.32
ResNetRS100	95.13	94.97	96.23	96.63	96.30	97.12	97.81	97.57	98.40
InceptionResNetV2	93.88	95.12	96.25	95.79	96.51	97.62	98.41	98.30	98.90
InceptionV3	94.38	94.45	95.79	95.55	95.60	97.01	98.03	97.97	98.67
Xception	93.30	93.20	94.94	95.51	95.59	97.01	97.75	97.80	98.54
MobileNetV2	91.21	91.10	93.09	93.66	94.61	96.27	98.13	97.94	98.65
EfficientNetV2B3	88.50	87.24	90.41	93.90	93.41	95.57	97.79	97.71	98.50
EfficientNetV2S	95.42	95.16	96.37	94.68	95.43	96.84	97.47	97.44	98.32
EfficientNetV2M	88.58	88.14	91.11	95.07	95.22	96.78	95.61	95.13	96.80
RegNetX008	94.38	94.66	95.99	94.60	95.31	96.81	98.59	98.52	99.03
RegNetY008	94.58	95.50	96.55	95.47	96.35	97.49	97.21	96.98	98.03

**Table 5:** The weight ratios of the CNN models in ensemble learning on three datasets.

DS1	Models	DenseNet121	DenseNet201	EfficientNetV2S	ResNet50	ResNet101
	Weights	0.209	0.212	0.237	0.038	0.304
DS2	Models	DenseNet121	DenseNet169	DenseNet201	InceptionResNetV2	ResNetRS100
	Weights	0.359	0.054	0.270	0.024	0.293
DS3	Models	DenseNet201	InceptionResNetV2	MobileNetV2	RegNetX008	ResNet101
	Weights	0.041	0.16	0.133	0.509	0.156

**Table 6:** Comparison of the proposed model with existing studies.

Study	Year	Dataset	Classes	Accuracy (%)	F1-Score (%)
Ayadi et al. [19]	2021	[56]	3	94.74	94.19 *
Deepak and Ameer [26]	2019	[56]	3	97.17	97.20
Ait-Amou [43]	2022	[56]	3	98.70	98.60
Kothandaraman [51]	2023	[56]	3	96.125	96.097
Wu and Sen [47]	2023	[56]	3	95.98	89.98
Alanazi et al. [35]	2022	[57]	4	95.75	95.72 *
Saurav et al. [24]	2022	[57]	4	95.71	95.98
Kang et al. [42]	2021	[57]	4	93.72	-
Aurna et al. [1]	2022	[58]	4	98.96	99.0
Gomez et al. [36]	2023	[58]	4	97.12	97.28
Proposed Model	2023	[56]	3	99.35	99.20
		[57]	4	98.77	98.92
		[58]	4	99.92	99.92

* Calculated from the given confusion matrix in the reference paper.

## Data Availability

Data are contained within the article.
